# Reduction Theories Elucidate the Origins of Complex Biological Rhythms Generated by Interacting Delay-Induced Oscillations

**DOI:** 10.1371/journal.pone.0026497

**Published:** 2011-11-07

**Authors:** Ikuhiro Yamaguchi, Yutaro Ogawa, Yasuhiko Jimbo, Hiroya Nakao, Kiyoshi Kotani

**Affiliations:** 1 Graduate School of Frontier Science, The University of Tokyo, Chiba, Japan; 2 Graduate School of Information Science and Engineering, Tokyo Institute of Technology, Tokyo, Japan; 3 CREST, JST, Kyoto, Japan; University of Maribor, Slovenia

## Abstract

Time delay is known to induce sustained oscillations in many biological systems such as electroencephalogram (EEG) activities and gene regulations. Furthermore, interactions among delay-induced oscillations can generate complex collective rhythms, which play important functional roles. However, due to their intrinsic infinite dimensionality, theoretical analysis of interacting delay-induced oscillations has been limited. Here, we show that the two primary methods for finite-dimensional limit cycles, namely, the center manifold reduction in the vicinity of the Hopf bifurcation and the phase reduction for weak interactions, can successfully be applied to interacting infinite-dimensional delay-induced oscillations. We systematically derive the complex Ginzburg-Landau equation and the phase equation without delay for general interaction networks. Based on the reduced low-dimensional equations, we demonstrate that diffusive (linearly attractive) coupling between a pair of delay-induced oscillations can exhibit nontrivial amplitude death and multimodal phase locking. Our analysis provides unique insights into experimentally observed EEG activities such as sudden transitions among different phase-locked states and occurrence of epileptic seizures.

## Introduction

In many natural systems, time delay can induce spontaneous breaking of continuous time translational symmetry and lead to self-sustained oscillations [1]–[4]. Such delay-induced oscillations play important functional roles in biological systems, e.g., human electroencephalogram (EEG) activities, Cheyne-Stokes respiration, ten-second oscillations of blood pressure known as the Mayer wave, mammalian circadian clocks, stem cell differentiation, and somite (body segments containing the same internal structures) segmentation in vertebrate embryos [3], [5]–[13]. In many cases, multiple biological oscillations interact with each other (e.g., in EEG activities and somite segmentation processes) and lead to complex spatio-temporal dynamics such as traveling waves and chaotic oscillations [5]–[7], [10], [14]–[19]. To understand these complex dynamics of biological systems, various mathematical models have been proposed [3], [6], [11]–[13], but much remains unknown about how complex dynamics arise from interactions of delay-induced oscillations.

In this study, we develop systematic reduction methods for coupled delay-induced oscillations. In analyzing dynamical models of biological systems, reduction methods are known to be considerably useful in facilitating mathematical treatments [16], [20]–[22]. The origins of biological complexity such as nonlinearity, noise effects, and complex network topology are elaborately incorporated and analyzed using the reduction methods [3], [21], [22]. So far, reduction methods for dynamical systems with delay have been mainly restricted to the situation that the time delay exists only in the interaction terms [23] and not fully utilized in the case that dominant time delay exists in each oscillator of the network because of its infinite-dimensional nature. In many biological systems, however, delayed feedback is the essential cause for the onset of oscillations, which should be properly taken into account. Therefore, a reduction theory for delay-induced oscillations, if successfully developed, would help elucidate how complex biological dynamics arise.

In the present paper, we give a systematic derivation of the reduced dynamical equations for coupled delay-induced oscillations. In particular, we analyze a cortico-thalamic model [24], [25] of EEG rhythms, described by coupled delay differential equations, by means of the center manifold reduction as well as the phase reduction methods. Using the reduced equations, we investigate the dynamics of the collective rhythms. In earlier studies, Campbell et al. [26]–[29] have performed detailed analyses of symmetrically coupled identical delay-induced oscillators by the center-manifold theory near the bifurcation point. However, biological rhythms are generally considered to arise from interactions of many oscillators, which can be heterogeneous and far from bifurcation points. Collective dynamics of such coupled delay-induced oscillators is still an open question. Our analysis would shed further light on their complex dynamics.

It has been argued that slow EEG rhythms are generated by mutual influence between the cortex and the thalamus with delays in transmission of electrical activities [24], [25], [30]–[34]. Because of the time delay, each local cortical area exhibits self-sustained oscillations. These local EEG oscillations interact with other areas of the cortex and constitute a spatially extended dynamical system, which can exhibit complex spatio-temporal patterns. To account for such EEG dynamics, Kim and Robinson proposed a cortico-thalamic model of EEG oscillations described by a second order differential equation with a linear time-delayed feedback [24], [25]. In the present study, we analyze a generalized system of Kim and Robinson's cortico-thalamic model, which describes a network of mutually interacting delay-induced oscillations, by two reduction methods. After deriving reduced equations for general networks, we focus on the simplest two-oscillator situation to elucidate unique properties of coupled delay-induced oscillations and reveal their nontrivial dynamics. In particular, we demonstrate amplitude death of delay-induced oscillations due to mutual coupling and multimodal phase-locking between delay-induced oscillations. We also show briefly that the reduction method can be successfully used to analyze nontrivial collective dynamics of a population of delay-induced oscillations. On the basis of our analysis, we argue the usefulness of reduction approaches in understanding biological rhythms.

## Results

### Model

Kim and Robinson's cortico-thalamic model of EEG oscillations was originally defined in spatially extended media [24], [25]. It was shown that the model reproduces several representative EEG behaviors such as slow waves, 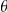 waves, and 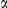 waves including epileptic seizures [25]. In the present study, we consider an extension of their model to general networks described by
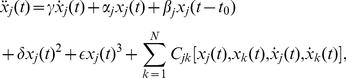
(1)where 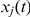 is a mean firing rate of neurons within each local area (denoted by 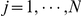) of the cortex [33], [34]; 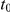 is the time delay; 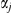 parameterizes the strength of cortico-cortical activities; 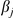 characterizes cortico-thalamic feedback; 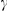 gives the damping rate; 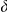 and 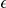 control the nonlinear terms that are originated from the characteristics of neuronal firing [24]; and the function 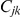 represents interaction between local areas 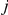 and 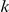. In this study, we take 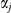 and 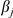 as variable parameters, while the other parameters are fixed at 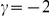, 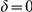, 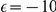, and 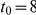. With these parameter settings, we can reproduce oscillations with various amplitudes and frequencies. In addition, setting 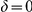 makes our analysis much simpler without loss of generality, as is reported by Kim and Robinson [24], [25].

In the absence of mutual interaction, each local area can exhibit stable delay-induced limit-cycle oscillations, i.e., it behaves as a self-sustained oscillator. We focus on the case that such oscillators are symmetrically and diffusively coupled with each other through the 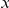 and 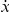 components; namely, the coupling function 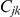 is given in the form
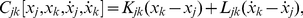
(2)where 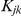 and 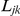 are coupling intensities of respective components between local areas 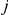 and 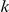 and specify the interaction network of this model. By this extension of Kim and Robinson's original model, we can take anisotropic and complex interactions of neural networks into account. The model described by Eqs. (1) and (2) goes back to the original model by Kim and Robinson [24] by considering regular spatial configurations of the oscillators, retaining only the coupling term 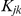, and taking a spatial continuum limit (with appropriate rescaling of time).

Though each individual oscillator has only a single variable, 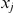, it has an infinite-dimensional phase space because of the time delay, which hampers detailed mathematical analysis. In the following, we will reduce each oscillator to a low-dimensional dynamical system and incorporate the interaction term as weak perturbations. Using the reduced coupled oscillator model with finite-dimensional phase space, we analyze the dynamics of mutually interacting delay-induced oscillations.

### Linear stability and reduced equations

The cortico-thalamic model Eq. (1) is described by coupled infinite-dimensional functional differential equations [35]. However, it is still possible, though technically more involved, to analyze the linear stability of the stationary state and apply reduction methods, similar to the case with low-dimensional limit-cycle oscillators.

#### Linear stability of stationary state

Let us first consider a single oscillator and analyze the linear stability of its stationary state. We omit the oscillator index 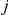 and drop the coupling function 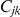 for the moment. It is clear that Eq. (1) has a stationary solution 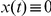. Let 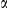 be a control parameter. Linear stability analysis with the ansatz 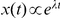 shows that this fixed point is destabilized when 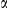 exceeds a critical value 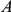 and that the critical eigenvalues are given by a purely imaginary and conjugate pair, 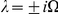, determined by a characteristic equation
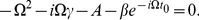
(3)Namely, each oscillator undergoes a Hopf bifurcation with frequency 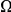 at 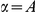. Because this bifurcation is supercritical under the condition 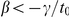 as will be shown by the center manifold analysis, we expect that a small amplitude limit cycle branches from the fixed point.

**Figure 1:**
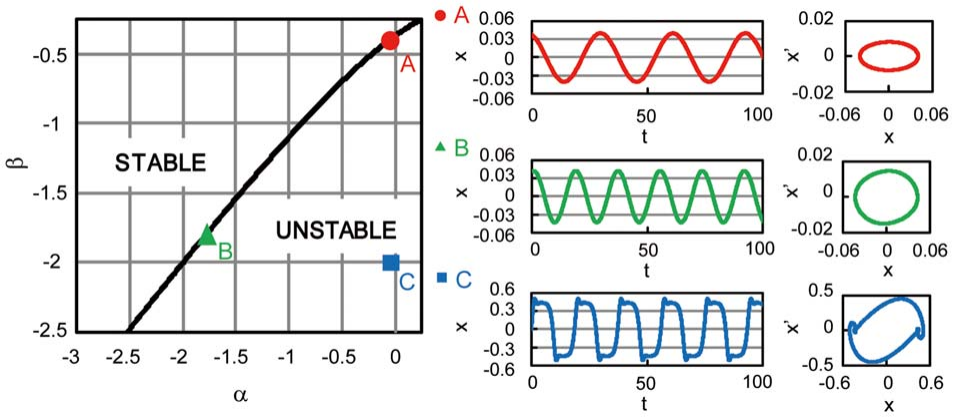
Stability region, time series and attractors of the cortico-thalamic model given by **Eq.(1)**. Left panel shows stable and unstable regions of a cortico-thalamic model without coupling, Eq. (1). The solid bifurcation curve is obtained from Eq. (3). Points A, B, and C represent the parameter sets used for the analysis [ A: 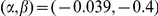, B: 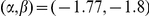 and C: 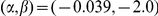 ], where the points A and B have the same 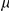 (equal distance from the stability boundary). Middle panels show the limit cycle oscillations and right panels their attractors at the parameter sets of A (top), B (middle), and C (bottom).

Figure 1 (left panel) shows the stability region of the stationary solution 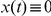 with respect to the parameters 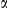 and 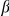. Let us decompose the parameter 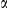 into the critical value 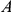 and a bifurcation parameter 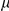 (i.e., 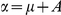). When the bifurcation parameter 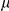 slightly exceeds 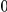, we observe sinusoidal oscillations of small amplitude, 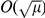. As 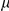 is increased further, the waveform of the oscillations becomes increasingly complex, reflecting the infinite dimensionality due to time delay. These characteristics are observed in time series and attractors shown in Fig. 1 (middle and right panels).

#### Center manifold reduction

Delay differential equations such as Eq. (1) should be considered as functional differential equations. The state of the system at time 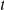 is described by a function on 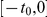, 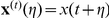 with 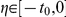. Therefore, the phase space in which the system state 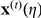 evolves is a function space of infinite dimension. Nevertheless, at the Hopf bifurcation point, 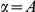, we can split the whole phase space into a low-dimensional center manifold and the remaining infinite-dimensional part, and derive a set of ordinary differential equations describing the reduced dynamics on the center manifold. This method is usually called the center manifold reduction. A center manifold reduction method for delayed dynamical systems is based on Hale's theory [35]. We apply the formulation developed by Campbell et al. [1] as well as by Wischert et al. [36] to simplify Eq. (1), which is conceptually similar to the conventional center manifold reduction for ordinary differential equations but technically more elaborate. We extend the analysis to coupled delay differential equations with small non-zero 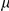 and incorporate the mutual interaction as perturbations. For the purpose of creating a stepping stone to more general cases, we focus on the case that the mutual interaction is sufficiently weak in the present study. This enables us to develop general mathematical framework for networks of interacting delay-induced oscillators. See Materials and Methods for the details of the derivation. The reduced coupled equations near the Hopf bifurcation point of the oscillators are given by
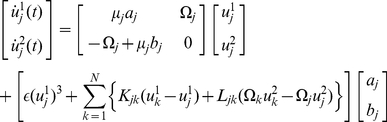
(4)for 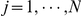, where 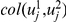 are the coordinates (or amplitudes) of the 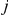-th oscillator on a two-dimensional center subspace. The superscript “1” or “2” indicates contravariant components, and does not mean the first or second power of 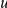. They are related to the original variables as 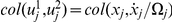 at the lowest order. Real values 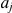 and 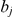 represent basis functions of the dual space of the center eigenspace (see Materials and Methods).

Furthermore, if we focus on the situation that the natural frequencies are narrowly distributed around 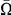 within the order of 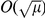, namely, 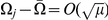, we can derive a further simplified equation from Eq. (1) by the averaging method [37] as
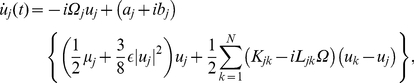
(5)where we introduced a complex variable 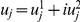. Note that the above reduced equations are finite-dimensional ordinary differential equations with only two dimensions for each oscillator, in contrast to the original equation (1) whose phase space is infinite-dimensional. The reduced model described by Eq. (5) is occasionally called (network version of) the complex Ginzburg-Landau equation and is widely studied in physics and applied mathematics [20], [21], [38].

#### Phase reduction

The phase reduction method, which describes a limit-cycle oscillator using only its phase variable by eliminating the amplitude degrees of freedom, can be adopted even if the oscillator is far from the bifurcation point. The necessary condition is that the oscillator has a stable limit cycle and is only weakly perturbed by the coupling. Although this method is usually applied to a finite-dimensional limit cycle, we can extend it to a limit-cycle solution of delay differential equations by appropriately defining the phase.

We first define an asymptotic phase 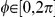 along the unperturbed limit cycle solution, which increases with a constant frequency rate 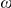 [20], [22]. This can easily be performed by measuring the time needed for the orbit to return to the phase origin, which we define as the point 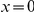 with positive 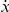 on the limit cycle. A fundamental quantity that characterizes the dynamical properties of a limit-cycle oscillator is the *phase response curve* (PRC), which gives the response of the oscillator phase to an impulsive stimulus [22], [39], [40]. When an impulse is applied at some phase point along the limit cycle, the orbit is kicked off the limit cycle and then relaxes to it again. During the course of this relaxation, the phase of the perturbed orbit deviates from that of the unperturbed orbit, and the asymptotic phase difference between these orbits gives the phase response of this limit cycle. Applying impulsive perturbations at varying phases of the limit cycle and observing the asymptotic phase responses, the PRC can be measured as a function of the phase.

For sufficiently weak perturbations, the PRC is proportional to the amplitude of the perturbation, so that the linear response coefficient, which we call *phase sensitivity*, is essential. In the present case, we measure the phase sensitivity function, 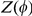, by perturbing the model (1). Once 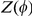 is obtained, we can calculate respective phase coupling functions 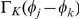 and 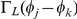 between the oscillators 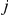 and 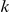 by convolving them with the time series of the coupling terms, 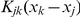 and 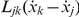, for one period of the limit-cycle oscillation [20], [22]. These functions describe effective phase interaction between weakly coupled oscillators and determine their dynamics. Assuming that all oscillators have similar characteristics, we can obtain the reduced coupled phase equations, which describe the phase dynamics of interacting delay-induced oscillations, as
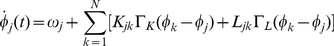
(6)for 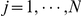. See Materials and Methods for the details.

When we consider two-oscillator systems with symmetric coupling [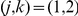 or 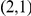 and 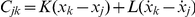], the dynamics of the phase difference 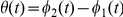 between the two oscillators can be derived from Eq. (6) as
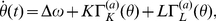
(7)where 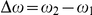 is the frequency difference and 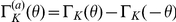 and 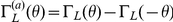 are anti-symmetric components of the respective phase coupling functions. Synchronization properties of the two oscillators can be immediately derived from this equation as shown in later sections.

### Validity of reduced equations near the onset of delay-induced oscillations

To confirm the validity of the reduced equations derived by the center manifold and the phase reduction methods, three comparisons were carried out around the point A in Fig. 1, 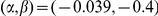, which is near the Hopf bifurcation of the delay-induced oscillations. We considered two-oscillator systems, 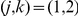 or 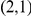.

**Figure 2:**
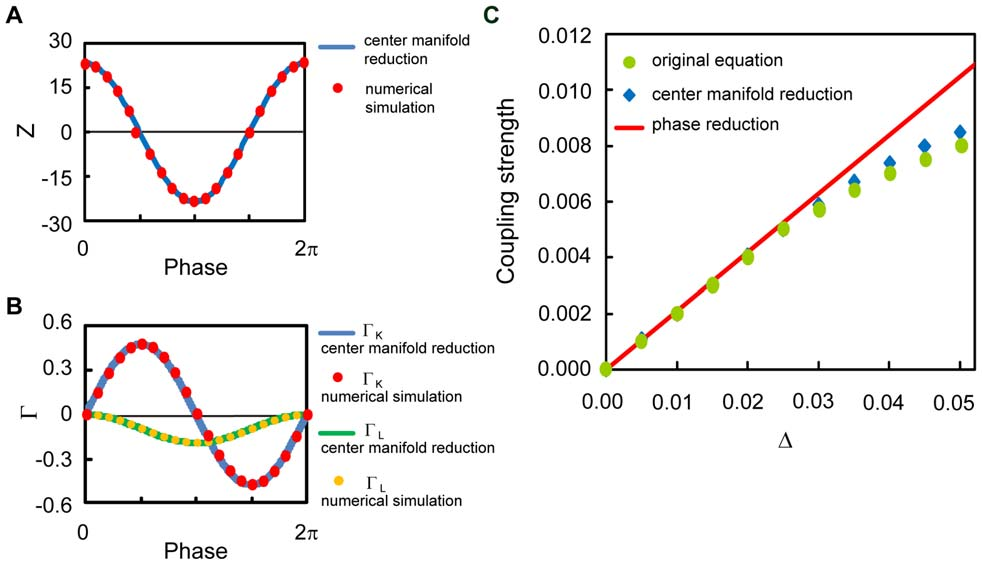
Numerically and analytically obtained synchronization properties of the coupled systems of delay-induced oscillations. (a) Phase sensitivity functions 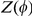 obtained analytically by center manifold reduction (solid line) and numerically by impulsive perturbation (dots). (b) Phase coupling functions obtained analytically by center manifold reduction (solid lines) and by numerical convolution (dots). (c) Critical coupling strength for synchronization of delay-induced oscillations. Green dots show the results of direct numerical simulations of the original delay differential equations, while blue dots indicate the results of numerical simulations of coupled amplitude equations obtained by the center manifold reduction. The red line is obtained from the phase coupling function (b).

First, the phase sensitivity function 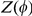 was obtained in two different ways: (i) numerical measurement by applying weak impulsive perturbations to Eq. (1), and (ii) analytical derivation by using the complex Ginzburg-Landau equation derived by the center manifold reduction, Eq. (5). Phase sensitivity functions of Eq. (5) with respect to the perturbations in the real and imaginary components of 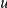 can be analytically derived as 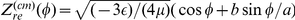 and 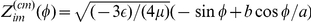, respectively [20]. Projecting the impulsive perturbation onto the center manifold, an analytical expression of 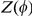 can be derived as
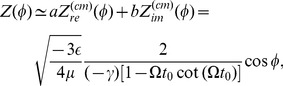
(8)which is sinusoidal reflecting the circular shape of the limit cycle near the Hopf bifurcation point of Eq. (1). Figure 2(a) compares 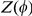 calculated in the two different ways, showing good agreement.

Second, the phase coupling functions 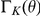 and 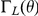 were also obtained numerically and analytically by convolving the coupling term 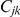 with the phase sensitivity 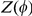 (Eq. (21) in Materials and Methods). Using Eq. (5), the phase coupling functions can be analytically calculated as
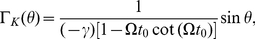
(9)and
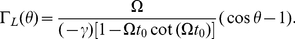
(10)These analytical results are compared with the numerical results in Fig. 2(b). Again, the analytical results and the direct numerical measurements are in good agreement for both of the phase coupling functions.

Third, critical values of the coupling strength for phase synchronization were compared near the point A for values obtained by (i) direct numerical simulations of the original delay differential equations (1), (ii) numerical simulations of the coupled amplitude equations (4) obtained by the center manifold reduction, and (iii) analytical calculations from the asymmetric part of the phase coupling function. We prepared two oscillators exhibiting equal-amplitude limit cycles with a small frequency mismatch and introduced weak mutual coupling only through the 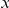-components as 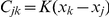 (see Materials and Methods). We did not include the coupling through the 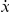-components because the phase coupling function 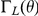 obtained in Eq. (10) is symmetric and does not affect the dynamics of the phase difference (see Eq. (7)). Figure 2(c) shows the critical coupling strength as a function of the frequency mismatch. The result based on the center manifold reduction is almost identical to that obtained by direct numerical simulations of the original model over the whole plotted range. Moreover, analytical calculations from the coupled phase equations (7) with the estimated phase coupling function 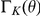 yield almost the same critical values for relatively weak coupling.

These results indicate that both the center manifold reduction and the phase reduction are appropriately accomplished near the point A in Fig. 1. Note that if the parameters 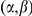 are far from the bifurcation point, the phase coupling functions can exhibit higher-harmonic fluctuations, reflecting complex limit-cycle orbits of delay-induced oscillations. This leads to an interesting multimodal phase locking behavior as we will demonstrate later.

We can also use the reduced phase equations to analyze collective behavior of a population of mutually interacting delay-induced oscillations. In the Supporting Information, we apply the phase reduction near the point A in Fig. 1 and demonstrate that the emergence of macroscopic synchronized state can be analytically predicted (see Text S1 and Fig. S1).

### Amplitude death of oscillations due to mutual coupling

It is known that relatively strong mutual coupling between limit cycles can induce amplitude death, a phenomenon which means disappearance of oscillations due to the stabilization of rest states [41], [42]. Here, we show that amplitude death, which has been observed in low-dimensional coupled limit cycles [41], also occurs in coupled delay-induced oscillations. Note that this phenomenon cannot be observed in coupled *phase* oscillators, i.e., when the mutual coupling is weak, because the amplitude degree of freedom is essential. We analytically predict the condition for amplitude death using the result of the center manifold reduction and verify it by direct numerical simulations of the original model.

**Figure 3:**
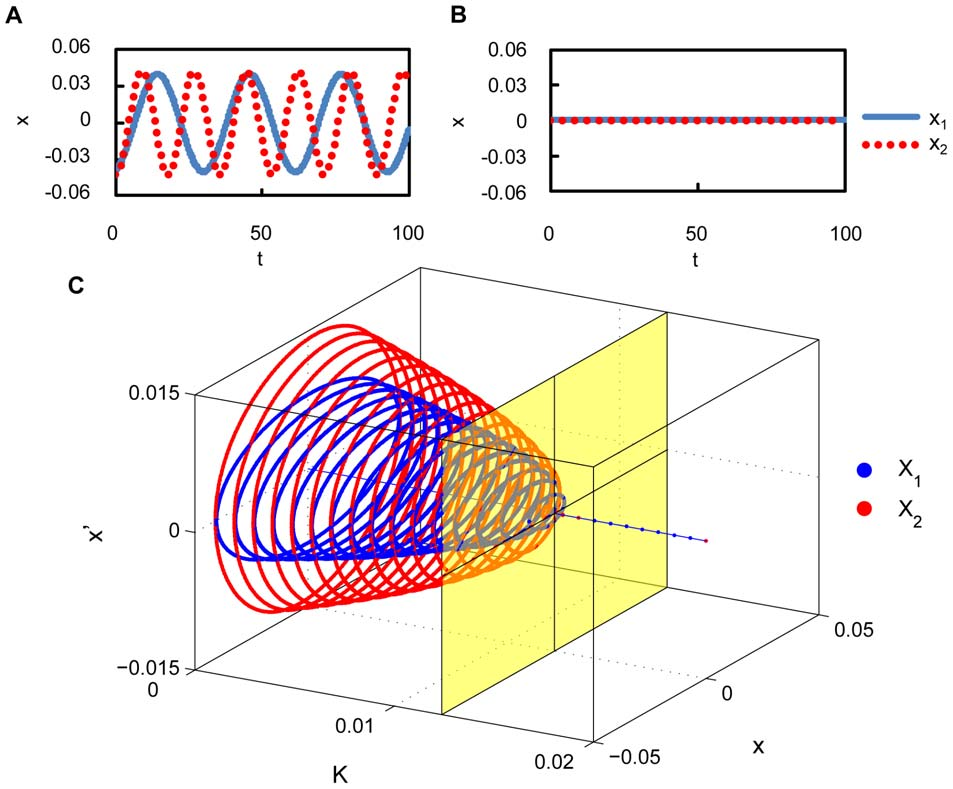
Amplitude death of the delay-induced oscillations due to mutual coupling. (a) Time series of delay-induced oscillations at the parameter sets A and B in Fig. 1 without coupling. (b) Amplitude death observed when the oscillators are coupled with 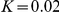. (c) Bifurcation diagram of coupled delay-induced oscillations. Blue and red points indicate the dynamics of the oscillators at parameter sets A and B, respectively. The light-yellow plane indicates 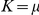. The oscillation disappears and the stable fixed point appears at the origin when 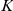 exceeds 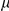.

Let us consider a coupled pair of oscillators whose parameters are at the points A and B in Fig. 1, respectively, interacting through the 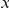-component, 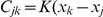). The points A and B have the same bifurcation parameter 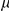, but the point B yields a larger value of 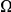. Thus, the oscillator B has a higher frequency than the oscillator A while their amplitudes remain the same (see Fig. 3a).

The condition for amplitude death can be derived from linear stability analysis of the fixed point of Eq. (4), 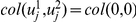 for 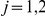. We can obtain the eigenvalues of the fixed point (see Materials and Methods) as
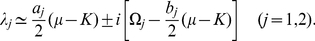
(11)Hence, if 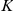 exceeds 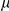, the real part of 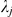 becomes negative, which implies the possibility of the amplitude death phenomenon.

Figure 3 shows the results of numerical simulations of the original model (1) with respect to the coupling strength 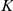. We can see that the oscillations actually die out when the coupling strength 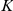 exceeds 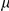. Therefore, it is confirmed that amplitude death actually occurs in systems of coupled delay-induced oscillations as predicted by the center manifold reduction.

The center manifold reduction can also be used to analyze the amplitude death effect in a population of coupled delay-induced oscillations that are distributed around the point A in Fig. 1. See Text S1 and Fig. S2 for details.

### Multimodal phase-locking

The most outstanding property of delayed dynamical systems is that even a simple equation can exhibit complex behaviors due to the systems' infinite dimensionality [3]. Here, we show that the phase reduction method can be applied to complex delay-induced oscillations with weak mutual coupling and leads to interesting quantitative predictions. In particular, we demonstrate phase synchronization with multiple stable phase-locking points, which cannot be observed in simple low-dimensional systems.

**Figure 4:**
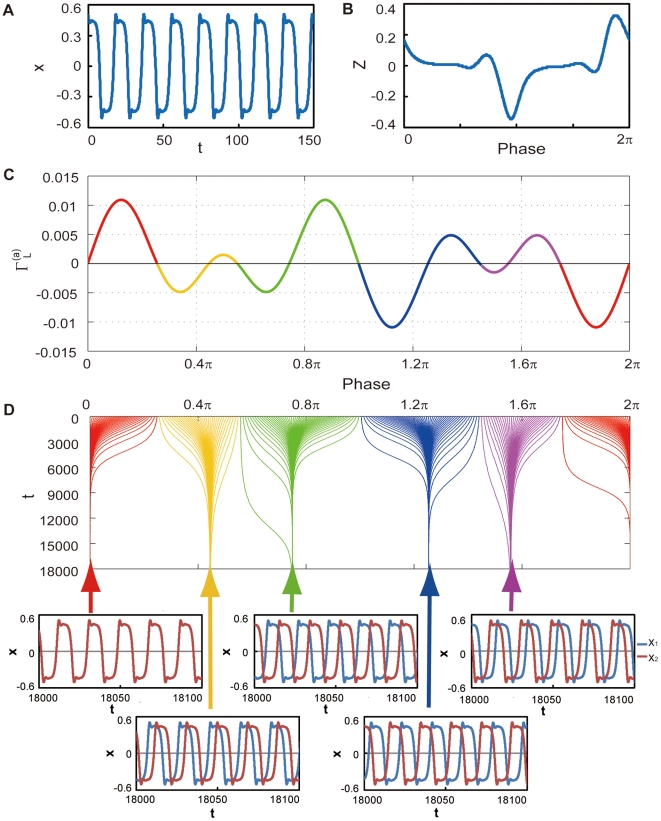
Multimodal phase-locking. (a) Limit cycle oscillation at the parameter set C in Fig. 1. (b) Phase sensitivity function 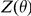 of the limit-cycle oscillation shown in (a). (c) Asymmetric part of the phase coupling function 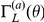. Each of the five colors indicates the predicted basin that converges to the same stationary phase difference. (d) Initial and transient phase differences for multimodal phase-locking of the coupled identical oscillators with 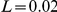. Initial phase differences plotted by the same color converge to the same stationary phase difference.

We consider a coupled pair of two identical oscillators at the point C in Fig. 1 coupled through the 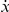 component, 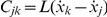 for 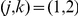 or 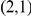. Each oscillator at this parameter is far from the bifurcation point, and therefore the center manifold reduction is no longer appropriate. The phase reduction is, however, still applicable because the oscillator exhibits a stable limit cycle as shown in Fig. 4(a) for weak mutual coupling. Dynamics of the phase difference between the oscillators are derived from Eq. (7) with 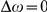 and 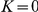. When 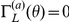 and 
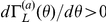
 at some 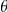, the two oscillators can be phase-locked at this 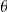.

The phase sensitivity function 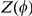 obtained numerically by applying small impulses to the unperturbed oscillator is shown in Fig. 4(b), and the asymmetric part of the phase coupling function 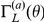 calculated from this 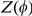 as well as the time series of 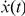 for one period of the limit cycle is shown in Fig. 4(c). Reflecting the relatively complex waveform of the delay-induced limit cycle, 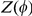 at the parameter C is not simply sinusoidal but exhibits higher-harmonic fluctuations. In Fig. 4(c), we can identify five points that satisfy 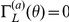 and 
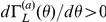
, implying that phase synchronization with five different values of the stable phase shift is possible. From this figure, we can predict five basins of the initial phase difference that will eventually synchronize at each value of the stable phase shift.

In order to confirm the above prediction based on the estimated phase coupling function, we performed direct numerical simulations of the original model Eq. (1) with fixed coupling strength 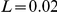 from varying initial phase differences. Figure 4(d) shows the transient dynamics of the phase differences, where uniformly distributed initial phase differences converge to the five stable points perfectly following the prediction of the reduced phase equations. Thus, the phase reduction approach works nicely for delay-induced oscillations and provides interesting results.

## Discussion

In this study, we applied center manifold and phase reduction theories to a system of interacting delay-induced oscillations. We could successfully reduce the dynamics of the system to low-dimensional coupled ordinary differential equations without delay, namely, the coupled amplitude equations (5) and the coupled phase equations (6). Generally, analytical treatments of dynamical systems with delays had been restricted due to their infinite-dimensional nature. We demonstrated that the two principal reduction methods can provide analytical predictions for interacting delay-induced oscillations. We successfully derived the network version of the complex Ginzburg-Landau equation and the phase equation known in physics and applied mathematics and whose dynamics can be studied in detail. We then showed that the effect of external perturbations and couplings are well described by reduced equations that illustrate validity of the reduced equations. Furthermore, synchronization properties of identical and nonidentical oscillators were appropriately evaluated by the complex Ginzburg-Landau equation near the bifurcation point as well as by the phase equation for weak coupling regimes (Figs. 2 and S1). These results give us analytical understanding of the collective dynamics of coupled delay-induced oscillations, even with inhomogeneity, which is necessary to understand real biological rhythms. Thus, our results will provide a useful starting point for understanding and controlling complex behaviors exhibited by interacting delay-induced oscillations.

In the cortico-thalamic model that we have analyzed, the variable 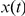 corresponds to scalp EEG voltages, which represent population dynamics of a number of neurons in the cortex [33]. For example, it is known that epileptic seizures are pathological dynamical states in which the neurons in the cortex are excessively synchronized, and suppression of such excess synchronization is an important problem in clinical medicine. It is experimentally demonstrated that deep brain stimulation (DBS) in which high frequency electrical stimulation is administrated to a sub-cortical target area can modulate the seizure threshold effectively [43]. However, detailed mechanisms of how DBS works are still unknown. The target area of DBS includes the thalamus and subthalamic nucleus that affects the thalamus via the substantia nigra pars reticulata. Therefore, we should investigate a cortico-thalamic model, rather than merely cortical models, for better understanding of the mechanisms of DBS. Detailed analysis of the reduced equations derived from the cortico-thalamic model may provide insights into electrical activities in the brain.

Our results on the amplitude death phenomenon suggest that interactions between delay-induced oscillations with different frequencies may affect the oscillations' stabilities and modulate their amplitudes. In the case of EEG, oscillations with various frequencies ranging from a few Hz (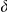 wave) to up to 80 Hz (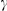 wave) are observed. Therefore, interaction between EEG oscillations with different frequencies might significantly affect the stability of their oscillations. This implication is supported by the report that EEGs of epileptic patients changes their properties preceding the onset of seizures (i.e., synchronization) over a wide frequency range [17]. Further studies on the cortico-thalamic model with the help of the reduction methods for interacting delay-induced oscillations might provide useful insights into the interactions of wide frequency oscillations in EEG, including epileptic seizures.

Regarding the phase reduction approach, the PRCs of delay differential equations have been measured in several studies, e.g., for models of circadian rhythms [44]–[46]. However, the collective dynamics of interacting delay-induced rhythms have not been studied in detail with this approach. Our present study demonstrates the utility of phase reduction for analyzing coupled systems of delay-induced oscillations. In particular, we revealed interesting multimodal phase-locking between delay-induced oscillations, which seems to be one of the outstanding features in interacting delay-induced complex oscillations far from the bifurcation point. Complex oscillations with higher harmonics similar to that we found at the parameter C in Fig. 4(a) are also observed in other models of nonlinear phenomena and sometimes called mixed-mode oscillations [47], [48]. Our results show that the time delay induces such complex mixed-mode oscillations and yields complex functional forms of 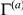, which leads to multimodal phase-locking.

The result of multimodal phase-locking implies that signals in different regions of the brain may easily change their phase relationships with others, even without transmission delays in the mutual coupling. For example, Roelfsema's experiment [15] showed that synchronization between cortical electrical activities suddenly alters between the in-phase locked state and the phase-locked state with a 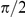 phase difference. Ito also reported similar sudden alternations in phase synchronization of human EEG signals [14]. Our result suggests the possibility that such alternations are caused by noise-induced transitions between different multimodal phase-locking states.

It is widely known that real neuronal networks have delays in synaptic and axonal transmission, and mathematical models with delayed coupling have been extensively analyzed [26]–[29], [49]–[54]. In contrast, we focused on the interacting slow rhythms of the EEG that arise when interaction delays between the cortex and the thalamus are dominant (and thus when coupling delays can be negligible). We considered interacting oscillators with different characteristics and derived coupled amplitude and phase equations for general networks. Although we mainly focused on two-oscillator cases as illustrative examples, the reduced equations are also applicable to the population dynamics of delay-induced oscillations (Text S1 and Figs. S1 and S2) even with general networks. Further studies on them will provide a wealth of interesting spatio-temporal dynamics of interacting delay-induced oscillations.

For example, we may consider arrays of neuronal populations with local interactions between neighbors using the same reduction methods. The reduced equations would typically exhibit wave propagation phenomena, which may explain some features of the EEG dynamics observed in the brain. Moreover, taking into account detailed neuronal network structures as well as transmission delays within a population [52]–[55] would also lead to interesting complex dynamics and may provide deeper understanding of the brain dynamics.

There are many biological systems in which self-sustained oscillations arise from delayed feedback and mutually interact with other oscillations. Therefore, we believe that our analysis will lead to a deeper understanding of various biological oscillations with delay, such as EEG dynamics (e.g., epileptic seizure and information processing between cortical regions), blood pressure regulation, and somite segmentations in vertebrate embryos.

## Materials and Methods

### Center manifold reduction

To carry out center manifold reduction of Eq. (1) in the vicinity of the Hopf bifurcation, we assume that all oscillators are close to the bifurcation point and the deviations of their bifurcation parameters from the critical value 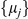 are small, i.e., 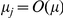. To extend the center manifold reduction to the neighborhood of the bifurcation point, we define three dynamical variables (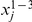) including the bifurcation parameter 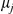 as
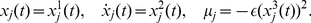
(12)Equation (1) with diffusive (i.e., linearly attractive, as given below) coupling terms given in Eq. (2) can be expressed with these three dynamical variables as
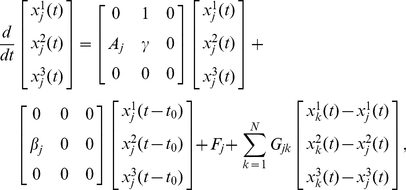
(13)where 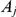 is the critical value of the parameter 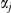 (i.e., 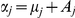), 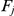 represents the nonlinear terms given by
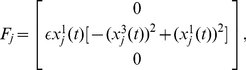
(14)and 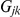 is a matrix of the coupling constants given by
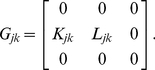
(15)From Eq. (13) with Eq. (14), it is clear that 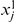 balances with 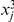 when the system exhibits limit-cycle oscillations because 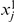 is of the same order as 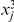. Following Hale [35], we define 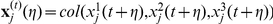 in the infinite-dimensional phase space and decompose it into the center eigenspace component and its complement as
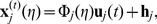
(16)where 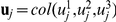 represents coordinates in the three-dimensional center eigenspace. Then, we obtain the basis functions of the center eigenspace as
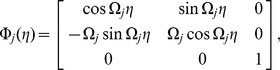
(17)and the corresponding dual basis as
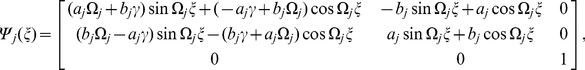
(18)where 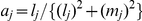, 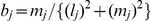 with 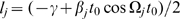, 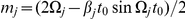. When 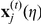 is on the center manifold, the order of 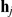 is 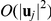. The reduced low-dimensional equation for 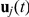 can be derived in the form
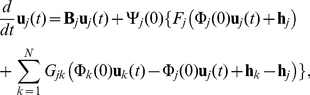
(19)where
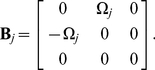
(20)Assuming that the coupling intensities are of the same order as 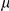, (i.e., 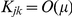 and 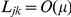), we can obtain the center manifold equation up to the third order in 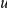. Fortunately, we do not need to calculate 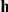 because it vanishes in the third-order equation when 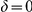. Returning to 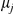 again from 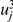, we arrive at the reduced amplitude equation given as Eq. (4). Using Eq. (4), we can analyze the system much more easily, because it does not contain delay terms anymore.

We can further bring the above equation into the complex Ginzburg-Landau equation when the differences in 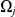 are narrowly distributed within the range of 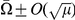. Adopting the averaging method [37], we can derive a simple symmetric form as given in Eq. (5). This complex Ginzburg-Landau equation clearly indicates several important characteristics of Eq. (1). First, the Hopf bifurcation is supercritical in the region shown in Fig. 1 because 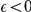 and 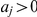 when 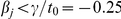. Second, this supercritical Hopf bifurcation yields a circular limit cycle of 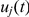 whose amplitude is 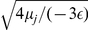, which is approximately equivalent to that of 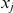 in this case (see Eq. (16)), and whose oscillation period is 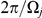. Third, the relaxation time constant of a perturbed orbit to return to the limit-cycle attractor is characterized by 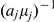. These predictions are confirmed in our numerical calculations of the original equation near the bifurcation point.

### Phase coupling functions

The phase sensitivity function 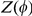 with respect to perturbations in the model (1) can be obtained analytically from the complex Ginzburg-Landau equation or numerically from direct numerical simulations of the original model. The time series of the coupling terms 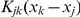 and 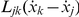 can also be obtained analytically or numerically for one period of the limit-cycle oscillation, 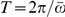, where 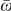 is the mean frequency of the two oscillators. The phase coupling functions can be calculated as the convolutions of the phase sensitivity functions and the coupling terms [20] as
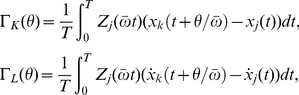
(21)which describe effective phase interactions between the two oscillators.

### Critical coupling strength

We prepare two oscillators with the same amplitude but whose frequencies are slightly different, and we then calculate the critical coupling strength for the phase synchronization. It is generally not easy to find appropriate values for parameters of the delay differential equation that satisfy such a condition, but in the present case, it is easily achieved using the results of the center manifold reduction ((Eqs. (3),( 5), and ( 16)). We choose the distances of the two oscillators from the respective bifurcation points as 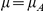, and their frequencies as 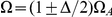, where 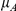 and 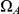 are the bifurcation parameter and frequency at the point A and 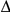 is the degree of frequency mismatch. The parameter sets (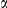, 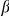) satisfying the above conditions are inversely determined from Eq. (3).

The critical values of the coupling strength for the synchronization are obtained by three methods, (i) direct numerical simulations of the original delay differential equations (1), (ii) numerical simulations of the coupled amplitude equations (4) obtained by the center manifold reduction, and (iii) analytical calculations using the asymmetric part of the phase coupling function, 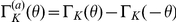, where the maximum value of 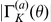 gives the slope of the critical coupling strength (Fig. 2(c)). In numerical simulations, we determined the synchronization transition by comparing the frequencies of the oscillators averaged over 1,000 rotations.

### Theoretical analysis for amplitude death

The condition for amplitude death can be derived from linear stability analysis of the fixed point of Eq. (4), 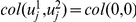 for (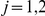). The matrix corresponding to the linear part of Eq. (4) under the condition of 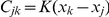 for 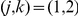 or 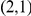 can be decomposed as
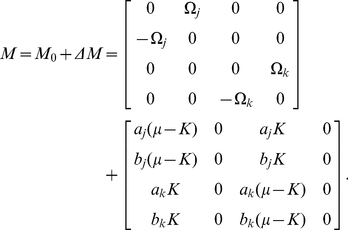
(22)The left and right eigenvectors of 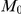 turn out to be
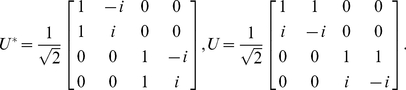
(23)Then, the perturbed eigenvalues Eq. (11) can be obtained from the diagonal components of 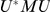.

## Supporting Information

Text S1 **Population dynamics of delay-induced oscillations.**
(PDF)

Figure S1
**Kuramoto transition in a population of delay-induced oscillations.** A: Distribution of the parameter sets 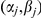 used in the numerical simulations. B: Time series of 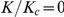 (top) and 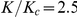 (bottom). The 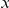 components of 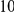 representative oscillators (chosen randomly) are plotted. C: The order parameter 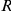 vs. the coupling strength 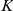. Macroscopic coherent rhythms emerge at 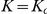.(TIF)

Figure S2
**Amplitude death in a population of delay-induced oscillations.** A: Distribution of the parameter sets 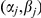. B–D: Snapshots of all oscillators (top) and time series of 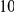 representative oscillators (bottom) at different values of the coupling strength 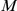 (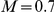 in B, 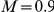 in C, and 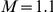 in D). The amplitude death occurs when 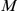 exceeds 1.(TIF)
