# Supplementary material for: Reduction Theories Elucidate the Origins of Complex Biological Rhythms Generated by Interacting Delay-Induced Oscillations
Source: PLoS One. 2011 Nov 7;6(11):e26497. doi: 10.1371/journal.pone.0026497 (PMC3210122; doi:10.1371/journal.pone.0026497)
Supplement: Text S1 — Population dynamics of delay-induced oscillations. (PDF) [file pone.0026497.s001.pdf]

I. SUPPORTING INFORMATION: POPULATION DYNAMICS OF DELAY-INDUCED OSCILLATIONS

In the main text, we focused on two coupled delay-induced oscillations and studied their dynamics using the two reduction methods. Here, to see the collective dynamics of many interacting oscillations, we analyze a population of delay-induced oscillations with heterogeneous frequency distributions using the reduced equations obtained in the main text. As the simplest case, we assume all-to-all global coupling among the oscillators and demonstrate the emergence of macroscopic synchronization and amplitude death phenomena in the population [1, 2]. They have been predicted to occur in systems of globally coupled limit cycles, but, to the best of our knowledge, this is the first demonstration in a system of coupled delay-induced oscillations. Both of the non-trivial collective dynamics can be analytically treated using the two reduction methods developed in the main text.

Since we consider global coupling, we assume $K_{jk} = K$ and $L_{jk} = L$ for all j and k in Eq. (2). The number of the oscillators used in the numerical simulations is $N = 256$. The mean frequency of the oscillators is chosen as the frequency obtained at the point A in Fig. 1, which we denote by Ω. The frequency of each oscillator is independently chosen around this Ω from a normal distribution with a fixed standard deviation σ. For all oscillators, we assume the same value of the control parameter μ, i.e., their distances from the bifurcation points are the same as that of the point A. The parameter sets $(\alpha_j,\ \beta_j)$ satisfying such conditions are then inversely determined from Eq. (3) and from the relation $\alpha = \mu + A$, while keeping other parameters fixed at the same values as those in the main text, i.e., $\gamma = -2, \delta = 0, \epsilon = -10$, and $t_0 = 8$.

A. Kuramoto transition in a population of delay-induced oscillations

When the mutual coupling is weak enough, oscillators with nonidentical frequencies behave incoherently and no macroscopic oscillations are observed. As the coupling strength is increased, the oscillators tend to synchronize with each other and macroscopic coherent rhythms emerge. The most well-known example is the macroscopic synchronization transition in systems of globally coupled oscillators investigated by Kuramoto [1]. Suppose a

system of N coupled oscillators described by the following phase model:

$$\dot{\phi}_j(t) = \omega_j + \frac{K_p}{N}\sum_{k=1}^{N}\sin(\phi_k - \phi_j),$$

where ϕ_j is the phase of the jth oscillator, ω_j is the natural frequency, and K_p is the coupling strength. The natural frequency ω_j of each oscillator is independently drawn from a probability density $g(\omega)$, which is assumed to be symmetric and unimodal with a peak at $\omega = \omega_0$. It can be shown that this system undergoes a macroscopic synchronization transition at $K_p = 2/(\pi g(\omega_0))$ and exhibits collective rhythms.

We here consider the Kuramoto transition in a system of globally coupled delay-induced oscillations described by Eq. (1) in the main text. When the frequencies of the oscillators are narrowly distributed and the coupling strength is relatively weak, we can approximate Eq. (1) by the reduced phase equation (6) and treat the collective dynamics of the system analytically. We assume that the oscillators interact only through the x component, i.e., we assume $K > 0$ and $L = 0$, and vary K as the control parameter. Equation (6) can then be converted to the Kuramoto model given above. The critical coupling strength K_c is predicted in Ref. [1] to be

$$K_c = \frac{2\sqrt{2}\sigma(-\gamma)(1 - \Omega t_0 \cot(\Omega t_0))}{\sqrt{\pi}N}.$$

To check this prediction, we performed numerical simulations of Eq. (1). The parameter σ was set at $\sigma = 1.5 \times 10^{-3}$. Figure S1(A) shows the parameter sets (α_j, β_j) used in the simulations. Figure S1(B) shows the time series of some of the delay-induced oscillations for $K < K_c$ (top panel) and for $K > K_c$ (bottom panel). We can clearly see that the oscillations are mutually synchronized when $K > K_c$, though their waveforms are not completely the same because their parameters are slightly different. Degree of the macroscopic synchronization is measured by the order parameter $R = \langle \frac{1}{N} | \sum_{j=1}^{N} x_j | \rangle$, where $\langle ... \rangle$ denotes time averaging. If all oscillators are perfectly synchronized at the same phase, $R = \sqrt{2\mu/(-3\epsilon)} \simeq 0.028$ is predicted from the reduced phase equation. Figure S1(C) shows the order parameter R with respect to the normalized coupling strength K/K_c. We can clearly observe the sudden increase in the order parameter R at $K/K_c = 1$, which indicates emergence of collective oscillations at $K = K_c$.

B. Amplitude death in a population of delay-induced oscillations

When the frequency distribution of the oscillators is relatively wide, the amplitude death phenomenon, in which all oscillators are stabilized at their origin and stop oscillations, is expected. Matthews, Mirollo, and Strogatz [2] carefully investigated the collective dynamics of the CGL Eq. (5) and derived the conditions for the amplitude death. It can be considered a generalization of the amplitude death phenomenon for two-oscillator systems that we treated in the main text.

For simplicity, we consider a specific case that the ratio of the coupling strengths in the x and $\dot{x}$ components is kept constant, namely, we assume that K and L are given by

$$K = \frac{M\mu a^2}{N(a^2+b^2)},\ L = \frac{M\mu ab}{N\Omega(a^2+b^2)},$$

with M being a control parameter. Equation (1) can then be reduced to the CGL equation (5) with a coupling term $(M\mu a/2N)(u_k - u_j)$, which is the form investigated in Ref. [2]. It is predicted that, under a necessary condition

$$\sigma > \sigma_c = \frac{a\mu\sqrt{\pi}}{2\sqrt{2}},$$

the amplitude death occurs when the control parameter M exceeds 1.

We fix the parameter σ at $\sigma = 1.5 \times \sigma_c$ for the numerical simulations, which results in the parameter sets (α_j, β_j) shown in Fig. S1(A). Figures S2(B-D) show snapshots (top panel in each figure) and time series (bottom) of the delay-induced oscillations for several values of M. We can confirm that the oscillations cease and the amplitudes vanish when $M > 1$, namely, the amplitude death actually occurs in a population of delay-induced oscillations.

---

[1] Kuramoto Y, *Chemical oscillations, waves, and turbulence* (Springer, New York, 1984).

[2] Matthews PC, Mirollo RE, and Strogatz SH (1991), Dynamics of a large system of coupled nonlinear oscillators, Physica D **52**, 293-331 (1991).
