# Supplementary figures and images for: Reduction Theories Elucidate the Origins of Complex Biological Rhythms Generated by Interacting Delay-Induced Oscillations
Source: PLoS One. 2011 Nov 7;6(11):e26497. doi: 10.1371/journal.pone.0026497 (PMC3210122; doi:10.1371/journal.pone.0026497)

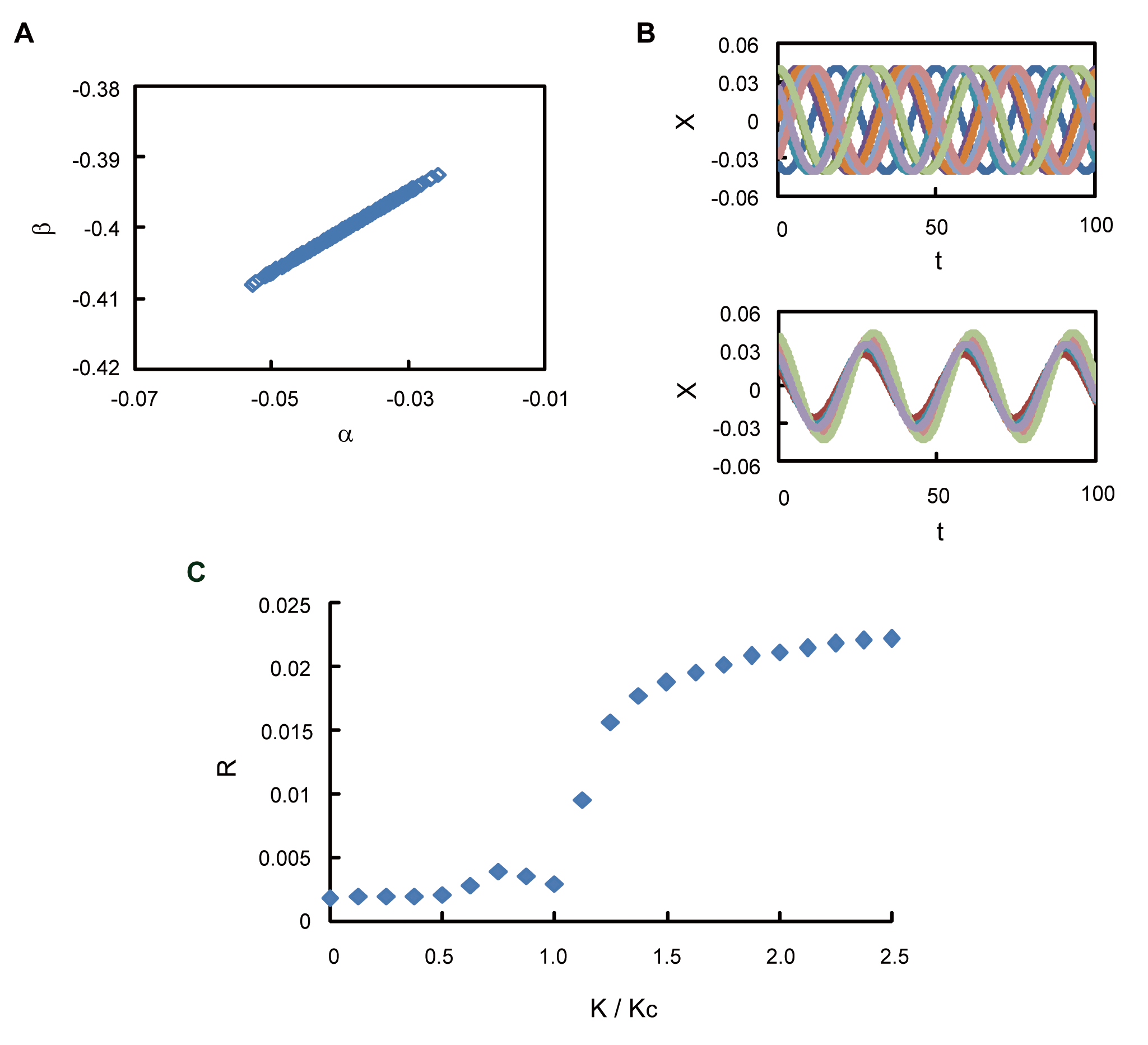

Supplement: Figure S1 — Kuramoto transition in a population of delay-induced oscillations. A: Distribution of the parameter sets used in the numerical simulations. B: Time series of (top) and (bottom). The components of representative oscillators (chosen randomly) are plotted. C: The order parameter vs. the coupling strength . Macroscopic coherent rhythms emerge at . (TIF) [file pone.0026497.s002.tif]

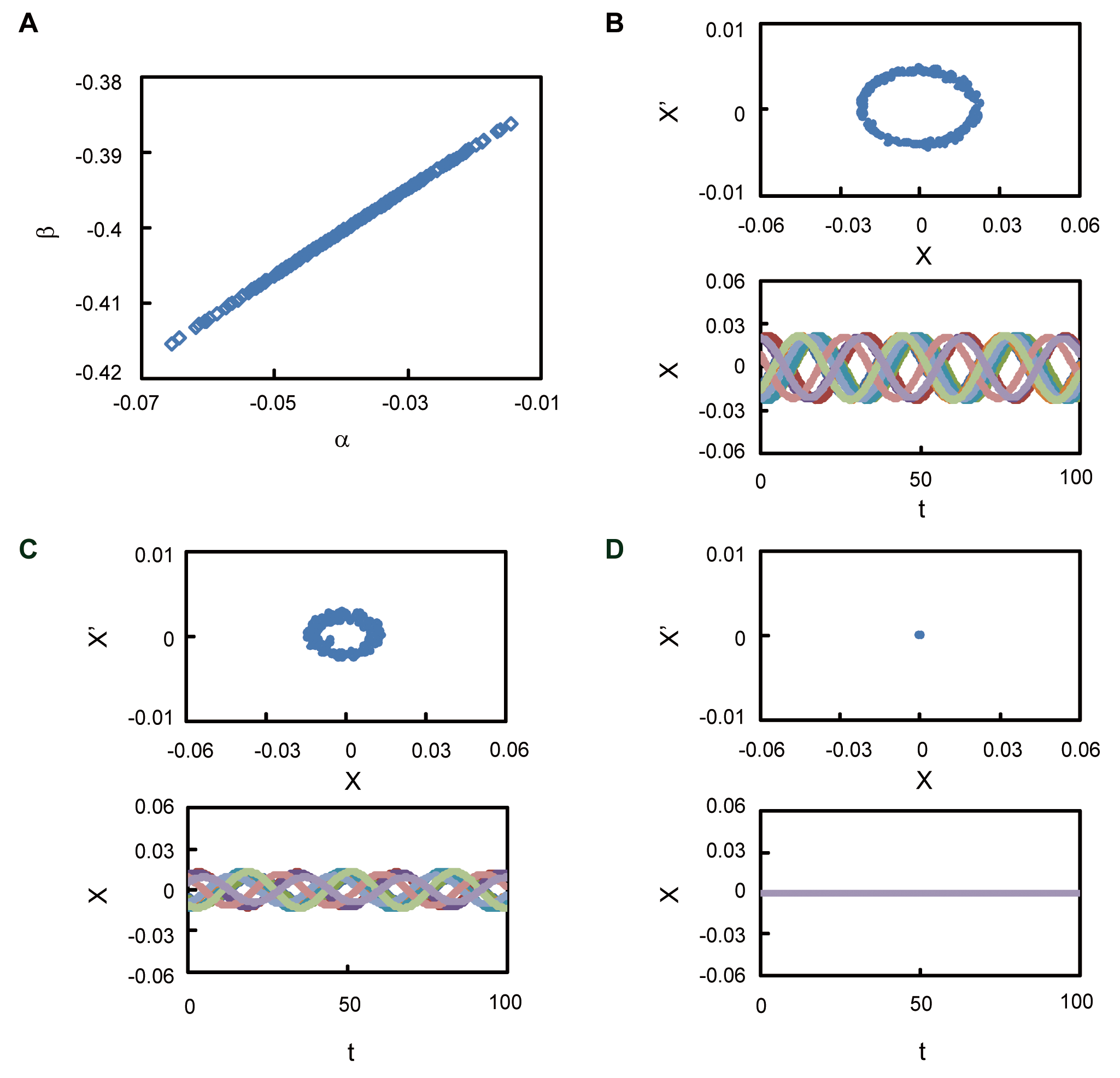

Supplement: Figure S2 — Amplitude death in a population of delay-induced oscillations. A: Distribution of the parameter sets . B–D: Snapshots of all oscillators (top) and time series of representative oscillators (bottom) at different values of the coupling strength ( in B, in C, and in D). The amplitude death occurs when exceeds 1. (TIF) [file pone.0026497.s003.tif]
